# Low-level colonization of methicillin-resistant *Staphylococcus aureus* in pigs is maintained by slowly evolving, closely related strains in Finnish pig farms

**DOI:** 10.1186/s13028-022-00653-y

**Published:** 2022-12-02

**Authors:** Marie Verkola, Milla Takala, Suvi Nykäsenoja, Satu Olkkola, Paula Kurittu, Saija Kiljunen, Henni Tuomala, Asko Järvinen, Annamari Heikinheimo

**Affiliations:** 1 Department of Food Hygiene and Environmental Health, Faculty of Veterinary Medicine, University of Helsinki, P.O. Box 66, 00014 University of Helsinki Helsinki, Finland; 2 Laboratory and Research Division, Microbiology Unit, Finnish Food Authority, P.O. Box 200, 00027 Finnish Food Authority Helsinki, Finland; 3 Human Microbiome Research Program, Faculty of Medicine, Helsinki University Central Hospital, University of Helsinki, P.O. Box 21, 00014 University of Helsinki Helsinki, Finland; 4 Department of Infectious Diseases, Inflammation Center, Helsinki University Central Hospital, University of Helsinki, P.O. Box 340, 00029 HUS Helsinki, Finland

**Keywords:** Antimicrobial resistance, cgMLST, LA-MRSA, Livestock-associated methicillin-resistant *Staphylococcus aureus*

## Abstract

**Background:**

Over the past two decades, livestock-associated methicillin-resistant *Staphylococcus aureus* (LA-MRSA) has become widely prevalent in pig production in Europe. The carriage status of LA-MRSA is known to vary among individual pigs, but bacterial load in pigs has rarely been studied. We assessed the quantity of LA-MRSA in nasal and skin samples of pigs and investigated the genetic diversity of the strains together with sequenced strains from national surveillance and pathology samples from the Finnish Food Authority. On two farms with assumed MRSA-positive status, farm 1 and farm 2, 10 healthy pigs were sampled three times during 2 weeks from the nares and skin (study A). On farm 1, 54 additional pigs were sampled and from confirmed MRSA-positive animals, 10 were randomly selected and transported to a clean, controlled environment for further sampling (study B). From the samples taken on farms 1 and 2 and in the controlled environment, MRSA was isolated both by direct plating and enrichment on selective media. *spa* types, multilocus sequence types, staphylococcal cassette chromosome *mec* types, resistance and virulence genes were determined. Core genome multilocus sequence typing (cgMLST) analysis was performed, including the sequences deriving from the surveillance/pathology samples from the Finnish Food Authority.

**Results:**

All pigs on farm 1 carried LA-MRSA in the nares at all three time points and five pigs on farm 2 at one time point. Nasal quantity varied between 10 and 10^3^ CFU/swab and quantity on the skin between 10 and 10^2^ CFU/swab. In the controlled environment, MRSA was detected in at least one of the nasal samples from each animal. *spa* type t034 was predominant. cgMLST showed one cluster with minimum allele differences between 0 and 11.

**Conclusions:**

The study shows predominantly low-level carriage (< 10^3^ CFU/swab) of LA-MRSA on farms. In the controlled environment we observed a decline in nasal carriage but constant skin carriage. cgMLST showed that strains of *spa* type t034 are closely related at the national level.

**Supplementary Information:**

The online version contains supplementary material available at 10.1186/s13028-022-00653-y.

## Background

Since the 1960s, problems with methicillin-resistant *Staphylococcus aureus* (MRSA) first touched health-care facilities, then the community and more recently livestock. In Europe, the livestock-associated MRSA (LA-MRSA) strains belong mainly to multilocus sequence type (ST)398 and clonal complex (CC)398 and were first discovered in livestock, especially pigs and veal calves [1, 2]. Numerous *spa* types are associated with MRSA CC398. *spa* types t011 and t034 are common in pigs in most parts of Europe, while other *spa* types are prevalent only in certain European countries or are found only sporadically [3–9]. LA-MRSA strains do not restrict themselves to animals and can be transmitted to humans. In particular, people working in close contact with MRSA-carrying animals are at risk of being colonized with LA-MRSA [10, 11]. Both animals and humans may serve as asymptomatic carriers. Carriage is a risk factor for clinical infection and may also lead to transmission to other animals or humans [12].

According to European Union (EU) legislation, monitoring of MRSA in food-producing animals is voluntary [13]. In Finland, based on a national decision, resistance of MRSA in fattening pigs and pork is monitored in occasional surveys. MRSA has not been surveyed comprehensively at farm level since the European Food Safety Authority baseline study in 2008 [prevalence 0.5% among fattening pig and breeding pig farms (n = 207)], and national prevalence data are only available from two slaughterhouse surveys. Both surveys were conducted as part of national surveillance in the five largest slaughterhouses, covering over 90% of pigs slaughtered in Finland. MRSA prevalence was assessed in pig slaughter batches taking nasal samples from five fattening pigs per slaughter batch at stunning. The surveys indicate an increase of LA-MRSA in pigs, with a 22% (13/59) prevalence in 2009–2010 compared with 77% (47/61) in 2016–2017 [14].

Pig production in Finland is concentrated in the south-western and western parts of the country. In 2016, there were 1240 pig farms, with a total of 1,235,000 pigs [15]. Measured in produced meat, pork production was the largest sector, with 190 million kg of pork produced in 2016 [16]. Between 100 and 200 pigs were imported yearly between 2008 and 2017 from Denmark, Norway, Sweden and Austria (Finnish Food Authority, unpublished data). Importation of live pigs from other European countries into Finland is thus very limited compared with many countries in continental Europe, where annual live pig imports range from hundreds of thousands to millions of pigs. Detection of LA-MRSA on a pig farm does not lead to any restrictions in Finland.

Among humans in Finland, the number of MRSA cases have been at a low level, with 1700 cases in 2016, 1435 in 2017 and 1391 in 2019 [17, 18], which gives annual incidences of 30.9 cases per 100,000 people, 26.0/100,000 and 25.2/100,000, respectively. However, the proportion of LA-MRSA CC398 of all MRSA cases increased during the years before the COVID-19 pandemic, being 2.9% in 2016, 3.4% in 2017 and 6.9% in 2019 [17, 18].

Carriage of methicillin-susceptible *S. aureus* (MSSA) and MRSA is mostly intermittent in individual pigs [19, 20]; thus reliable surveillance requires testing MRSA at herd level rather than in individual pigs [20] and the use of several sampling sites [21, 22]. Sampling both nares and the skin of the axilla, the perineum or behind one ear has proven effective [21, 22]. It seems difficult to establish whether individual pigs are truly colonized, i.e. bacteria actually multiply on the skin and/or mucous membranes, or whether they deliver positive samples due to contamination from other animals or the environment [20]. Studies have shown that dust in MRSA-positive pig farms is contaminated by MRSA [23] and that the intensity of air contamination correlates positively with nasal MRSA carriage rates in humans [24]. It has been suggested that only a small proportion of pigs with exceptionally high MSSA and MRSA nasal loads are persistently colonized and that these high-carrier pigs with nasal loads of at least 10^4^ CFU/swab and the contaminated environment help the bacteria to keep circulating in pig herds even though most of the pigs are only intermittently colonized [25]. To our knowledge, only one study has used quantitative methods to assess MSSA and MRSA colonization in pigs [25]. The study did not look into the molecular epidemiology of the MSSA and MRSA strains, which would have given insight into the genomic characteristics and the diversity or relatedness of the strains isolated. In addition, the quantities were not reported at the individual pig level and only one anatomical site (nares) was sampled.

The aim of this study was to assess quantities of LA-MRSA CC398 in both nasal and skin samples of pigs in the farm environment and a controlled, thoroughly cleaned and disinfected environment to gain new insights into the dynamics of LA-MRSA carriage in pigs. Our hypothesis was that LA-MRSA levels would decline when pigs were removed from the farm environment. Reducing the amount of LA-MRSA in pigs could help prevent transmission from pigs to humans. *spa* typing, whole genome sequencing (WGS) and core genome multilocus sequence typing (cgMLST) were used to further characterize antimicrobial resistance patterns and genetic relatedness of the strains through time.

## Methods

### Study design

**Fig. 1:**
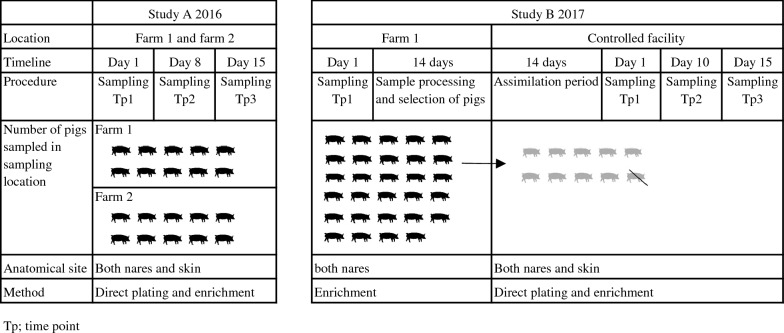
Timeline of study, sampling procedure and methods used for isolation of methicillin-resistant *Staphylococcus aureus* on farm 1, farm 2 and in the controlled facility

During 2016–2017, pigs were sampled in two studies in three different locations (Fig. 1). Study A was conducted in 2016 on two pig farms. Farm 1 was a farrow-to-finish farm of 700 sows (Norwegian Landrace) that had tested LA-MRSA-positive in a screening in 2015 (unpublished data) and farm 2 was a small farrow-to-finish farm with 120 sows (Norwegian Landrace) known to receive gilts from farm 1 and thus expected to be MRSA-positive as well. In case of infection, antibiotics were administered parenterally to individual pigs: amoxicillin or penicillin on farm 1 and sulphadoxine-trimethoprim, penicillin or amoxicillin on farm 2. Zinc oxide to prevent post-weaning diarrhoea was only used on farm 1. On both farms, weaning pigs of approximately 15 kg (n = 10) from one pen were chosen randomly for sampling: on farm 1 out of 30 pigs and on farm 2 out of 15 pigs.

In 2017, study B was initiated on farm 1 where a total of 54 weaning pigs from three pens were tested for MRSA (Fig. 1). From all MRSA-positive pigs, 10 pigs were randomly chosen and transported to the controlled environment of the large animal facilities of the University of Helsinki Laboratory Animal Center, referred to as the controlled facility in this article. The pigs originated from two neighbouring pens on farm 1. They were kept in one room in two pens with five pigs each and with constant contact between the two pens. The room had been properly cleaned and disinfected before the arrival of the pigs. All personnel and scientists wore disposable protective clothing throughout the trial period when entering the room, and everything taken out or into the rooms was disinfected. As one pig had to be euthanized due to injury three days after transport, the results could be reported for nine pigs only.

For the cgMLST, we also included the samples of the nine pigs taken at farm 1 when the pigs were chosen for the experiment. To compare the strains with other LA-MRSA strains isolated in Finland, sequences deriving from 17 LA-MRSA CC398 *spa* type t034 isolates from surveillance and pathology samples of the Finnish Food Authority taken from pigs between 2008 and 2017 were included in the cgMLST analysis. The sequences were a convenience sample of LA-MRSA t034 strains from different years with an emphasis on the years 2016 and 2017 when studies A and B were conducted. Ten of the isolates from the Finnish Food Authority collection were from surveillance samples: one was taken on a pig farm and nine from slaughtered pigs in the three largest slaughterhouses (Additional file 1). Two isolates originated from infection samples taken from pigs during pathological examination. Five isolates originated from screening samples taken on different pig farms. None of the isolates originating from infection or farm samples were taken from pigs originating from the study farms or on the study farms. As for the slaughterhouse samples, each isolate originated from a different slaughter batch and each slaughter batch represented a different farm. It is not known from which farms the slaughtered pigs originated.

The experiment at the controlled facility was approved by the Project Authorization Board ELLA (project identification code ESAVI/7280/04.100.07/2017). During the experiment a rigid biosafety protocol was followed. The pigs’ welfare was monitored throughout the study, as has been reported elsewhere [26].

### Sample collection

#### Sampling for study A

On both farms, 10 pigs from one pen were sampled from both nares and the skin three times at one-week intervals by the same person (Fig. 1). Nose swabs were taken from both nares with sterilized 15 cm cotton-tipped swabs (Selefa Ref. 120783, OneMed, Stockholm, Sweden). The swab was inserted into the nose and rubbed in a circle once. Skin swabs were taken with a cotton swab moistened with 0.9% saline solution behind one ear from an area of approximately 5 cm × 5 cm. Both swabs were then placed in separate test tubes with 1 mL of 0.9% saline solution.

#### Sampling for study B

For initial selection on farm 1, nose swabs were taken from both nares of 54 pigs with a cotton-tipped transport swab (M40 Transystem Amies Agar Gel, Copan Diagnostics, Brescia, Italy). At the controlled facility, nose and skin swabs were taken as described for study A three times within 15 days: on day 1, day 10 and day 15 (Fig. 1). Both nose and skin swabs were placed in separate test tubes each containing 1 mL of buffered peptone water (BPW) (Oxoid, Basingstoke, UK).

### Isolation of Staphylococcus aureus

#### Quantitative assessment of MRSA

Nose and skin swabs were kept cold at 4 °C until processing within 12 h (study A) and 2 h (controlled facility) of sampling. For direct plating, samples were thoroughly vortexed and serially diluted (10^−1^ to 10^−4^) with 0.9% saline solution or BPW. Next, 100 µL of the original sample and of each of the dilutions was pipetted onto CHROMagar MRSA plates and spread over the surface of the agar with a sterile spreader. After incubation at 37 °C for 18–24 h, the plates were read and separate typical mauve colonies were counted. Three typical colonies from the most diluted plate were streaked onto bovine blood agar and incubated for 16–24 h at 37 °C.

#### Qualitative assessment of MRSA

For enrichment of the samples of study A, 100 µL of the original saline solution was added to 900 µL of Müller Hinton broth (Oxoid, Basingstoke, UK) with 6.5% NaCl and incubated at 37 °C for 16–24 h. After incubation, a 10 µL loopful of the enrichment was streaked onto CHROMagar MRSA (CHROMagar Microbiology, Paris, France) and incubated at 37 °C for 18–24 h. The plates were read and 1–3 typical mauve colonies were streaked onto bovine blood agar and incubated for 16–24 h at 37 °C. Colony morphology was registered and plates with mixed growth were cultured again on bovine blood agar until pure growth was reached.

The control strains used to ensure proper detection of LA-MRSA with CHROMagar MRSA plates for the farm isolates in study A (2016) and for the controlled facility and farm isolates in study B are shown in Table 1.

**Table 1:** Control strains used in studies A and B

Bacterial strain	Clonal complex	*spa* type	*mec* gene
Study A			
Positive control strains			
15 SA-86^a^ *Staphylococcus aureus* (MRSA)	CC398	t034	*mecA*
AF 1214–14^a^ *Staphylococcus aureus* (MRSA)	CC398	t011	*mecA*
AF 980–14^a^ *Staphylococcus aureus* (MRSA)	Not known	t1657	*mecA*, *mecC*
AF 1582–14^a^ *Staphylococcus aureus* (MRSA)	Not known	t834	*mecC*
Negative control strains			
ATCC 8095^b^ *Staphylococcus aureus*	Not known	Not known	N.A
ATCC 8096^b^ *Staphylococcus aureus*	Not known	Not known	N.A
ATCC 25178^b^ *Staphylococcus aureus*	Not known	Not known	N.A
ATCC 6538^b^ *Staphylococcus aureus*	Not known	Not known	N.A
CH 19^c^ *Escherichia coli*	Not known	Not known	N.A
Study B			
Positive control strain			
AF 1214–14^a^ *Staphylococcus aureus* (MRSA)	CC398	t011	*mecA*
Negative control strains			
ATCC 12600^b^ *Staphylococcus aureus*	Not known	Not known	N.A
ATCC 25922^b^ *Escherichia coli*	Not known	Not known	N.A

*MRSA* methicillin-resistant *Staphylococcus aureus*; *NA* not applicable

^a^Source: National Institute for Health and Welfare

^b^Source: American Type Culture Collection

^c^Source: Isolated from Finnish wastewater

Due to mixed growth on the selective plates in study A, the two-step enrichment protocol of the EU Reference Laboratory for Antimicrobial Resistance [27] was applied to the samples taken on farm 1 in 2017. The protocol, including an additional enrichment step with cefoxitin and aztreonam, was followed with slight modifications—samples were enriched individually in 9 mL of Müller Hinton broth with 6.5% NaCl and incubated for 20 h at 37 °C and CHROMagar MRSA was used as the selective agar. One typical mauve colony was streaked from the selective agar onto bovine blood agar (Columbia blood agar base, Oxoid, Basingstoke, UK) and incubated overnight at 37 °C.

For the samples taken at the controlled facility, qualitative enrichment was started for each sample 1 week after sampling following the same protocol with one modification—for pre-enrichment, 3 mL of Müller Hinton broth with 6.5% NaCl was added to each test tube containing the sample and 700–800 µL of BPW.

All isolates were stored at − 70 °C on cryopreservation beads (Protect Microorganism Preservation System, Technical Service Consultants, Lancashire, UK).

#### Bacterial species confirmation and antimicrobial susceptibility testing

Isolates were cultured on bovine blood agar and incubated at 37 °C for 16–21 h. Morphology, catalase testing, Gram staining and coagulase testing with rabbit plasma (BD BBL coagulase plasmas, Franklin Lakes, NJ, USA) were performed. The same control strains as for CHROMagar MRSA were used during phenotypic confirmation.

For gram-, catalase- and coagulase-positive cocci, antimicrobial susceptibility of the isolates was tested using 30 µg cefoxitin discs (Rosco Diagnostica, Taastrup, Denmark) and interpreted according to European Committee on Antimicrobial Susceptibility Testing epidemiological cut-off values.

#### Confirmation of MRSA and spa typing

Template DNA was extracted from all catalase-, coagulase- and gram-positive cocci isolated in study A and at the controlled facility. A 1 µL loopful of bacteria from freshly cultured bovine blood agar was suspended in 100 µL of a solution containing 0.1 mg/mL lysostaphin (Sigma-Aldrich L7386, Merck, Darmstadt, Germany) and 1 mg/mL lysozyme (Sigma-Aldrich L4919) in a Tris buffer (50 mM Tris buffer (pH 8), 50 mM NaCl and 25% saccharose). The suspension was incubated at 37 °C for 60 min. Next, 50 µL of a solution containing 5 mg/mL proteinase K (Sigma-Aldrich P2308) in 50 mM Tris buffer (pH 8) was added and the suspension was incubated at 50 °C for 30 min. Proteinase K was inactivated through cooking for 5 min. After cooling, the suspension was centrifuged at 4 °C at 18,000 ×*g* for 10 min.

If multiple isolates existed from the same sample, only one was randomly picked. Confirmation of MRSA was done by in-house multiplex polymerase chain reaction (PCR) targeting *mec* using the method developed by Stegger et al. [28] following the protocol recommended by the EU Reference Laboratory [29].

For the farm isolates obtained in 2017, PBP2′ testing (PBP2′ Latex Agglutination Test, Oxoid, Basingstoke, UK) was performed according to the manufacturer’s instructions.

In addition, separate simplex PCR targeting the *spa* gene was performed for all strains isolated in the controlled facility and a selection of 42 strains isolated in 2016 using a method developed by Shopsin et al. [30]. From each pig sampled on farm 1, one MRSA isolate from each time point was chosen for PCR depending on availability in the following order: (1) nasal strain isolated through direct plating, (2) nasal strain isolated through enrichment, (3) skin strain isolated through direct plating, (4) skin strain isolated through enrichment. Nasal strains were preferred as they were thought more likely to represent the strain truly colonizing the pig sampled [22]. From farm 2, all seven isolates were selected. PCR products were sequenced at the University of Helsinki Institute of Biotechnology with Sanger sequencing, and *spa* types were deduced with BioNumerics 7.6.3 (Applied Maths, Sint-Martens-Latem, Belgium).

### Whole genome sequencing

#### DNA extraction

**Fig. 2:**
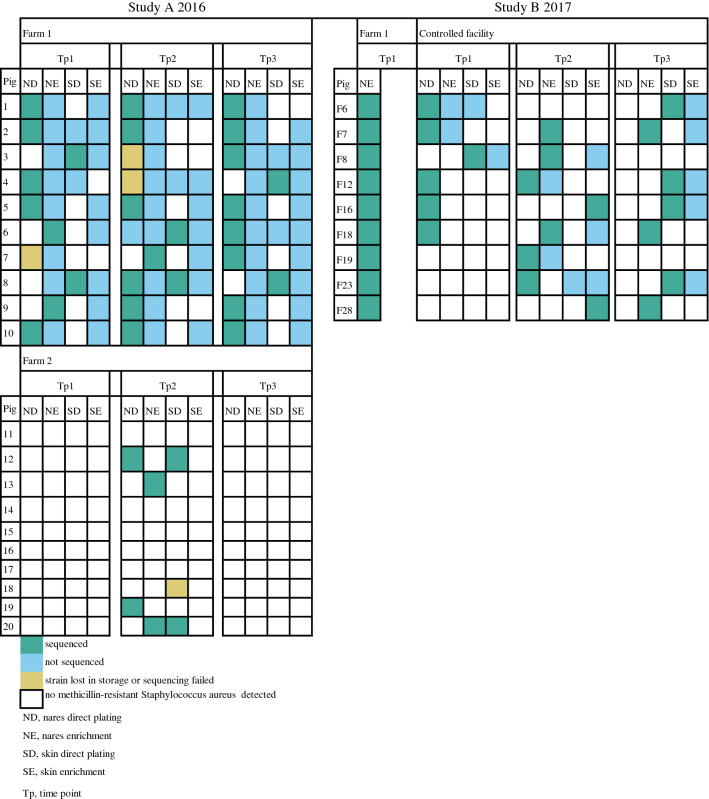
Retrieved isolates per pig per sampling time point, anatomical site and method of plating. Numbers refer to number of pig. Pigs in study B are additionally marked with the letter F

The strains for WGS were selected according to the same principles as for PCR (Fig. 2). DNA of *S. aureus* isolates from the farms and the controlled facility was extracted using the method described by Keto-Timonen et al. [31] with modifications. Strains were grown in 7 mL of Tryptone Soya Broth (Oxoid, Basingstoke, UK) at 37 °C for 16 h. Cells were harvested from 2 mL of culture, lysed in 400 µL of TE (10 mM Tris–HCl, 1 mM EDTA) with 100 µL lysozyme (final concentration 8.1 mg/mL), 100 µL mutanolysin (final concentration 161 IU/mL), 10 µL lysostaphin (final concentration 16.1 µg/mL) and 10 µL ribonuclease (final concentration 161.3 µg/mL) and incubated at 37 °C with gentle shaking for 2 h. For complete lysis, 9.2 mM of EDTA, 0.19 M of NaCl and 2 µL of proteinase K (final concentration 47.3 µg/mL) were added. After thorough mixing (vortex), 0.8% (v/v) sodium dodecyl sulphate was added. If the mixture did not turn opalescent after thorough mixing, 0.3% (v/v) sodium dodecyl sulphate was added. The mixture was incubated at 60 °C for 1 h under shaking. After phenol–chloroform–isoamyl alcohol and chloroform-2-pentanol extractions and ethanol precipitation, the DNA was left for thorough vaporization of ethanol at room temperature overnight. DNA was resuspended in TE buffer.

Strain 19A2 was cultivated in 5 mL of lysogeny broth overnight at 37 °C and 200 rpm. Cells were collected by centrifugation, and DNA was extracted using the NucleoSpin Microbial DNA Mini kit (Macherey–Nagel, Düren, Germany) according to the manufacturer’s instructions. For the sequences from the Finnish Food Authority, DNA of the strains was extracted with DNeasy Blood & Tissue kit (Qiagen, Hilden, Germany) using the gram-positive protocol according to the manufacturer’s instructions.

Quality control for DNA purity was performed with the NanoDrop ND-1000 (NanoDrop Technologies, Wilmington, DE, USA) and agarose gel electrophoresis, and concentration was measured with the Qubit 2.0 (Invitrogen, Thermo Fisher Scientific, Carlsbad, CA, USA) for the DNA of all other isolates except isolate 19A2, for which the Qubit 4.0 and agarose gel electrophoresis were used. Paired-end WGS was performed on the Illumina NovaSeq 6000 platform with a read length of 2 × 100 bp (Center for Genomics and Transcriptomics, Tübingen, Germany) (Finnish Food Authority strains and strains from study A with the exception of isolates 6A3, 8A2 and 19A2). The DNA of the isolates of study B and the farm isolates 6A3, 8A2 and 19A2 were paired-end sequenced on the Illumina NovaSeq 6000 platform with a read length of 2 × 150 bp (Novogene, Cambridge, UK).

#### Sequence analysis

Before the analysis, demultiplexing of the sequencing reads was performed with Illumina CASAVA 2.17. Adapters were trimmed with Skewer 0.1.116 [32]. The quality of the FASTQ files was analysed with FastQC 0.10.5-cegat [33] (strains from study A with the exception of isolates 6A3, 8A2 and 19A2). For the sequences from study B as well as strains 6A3, 8A2 and 19A2, demultiplexing was performed with bcl2fastq and adapter trimming and quality control were performed with fastp [34].

Raw reads for farm and controlled facility isolates were entered into the Center for Genomic Epidemiology (CGE) database spaTyper 1.0 [35] and SCC*mec*Finder 1.2 [36]. MLST [37] (dates accessed: 18 November 2020 and 20 May 2021), ResFinder [38] (dates accessed: 15 and 18 June 2021) and VirulenceFinder [39] (dates accessed: 18 November 2020 and 20 May 2021) were run locally using the script and databases in the repository of the CGE (https://bitbucket.org/genomicepidemiology/). The following settings were used: SCC*mec*Finder 1.2 and VirulenceFinder: threshold for %ID 90% and minimum length of coverage 60%, MLST threshold for %ID 95% and minimum coverage 60%, ResFinder minimum threshold for %ID 80% and minimum coverage 60%. For MLST and ResFinder, *S. aureus* was selected.

#### Core genome multilocus sequence typing

Raw reads of all isolates, including isolates from the Finnish Food Authority, were uploaded to SeqSphere + 7.0.4 (Ridom, Münster, Germany) and assembled by the automated pipeline of the software using FastQC 0.1.1.7 [33] for quality assessment, Trimmomatic 0.36 [40] for adapter trimming and SKESA 2.3.0 for de novo assembly [41]. For cgMLST, the default scheme of 1861 target genes was applied. To create a minimum spanning tree, columns with missing values were removed and a default cluster distance threshold of 24 was applied.

## Results

### Low levels of MRSA in nose and skin samples

Altogether, 89 isolates identified as MRSA were obtained from farm 1, 7 from farm 2 and 36 from the controlled facility (Fig. 2). All of these isolates proved positive for methicillin resistance mediating gene *mecA* in PCR and phenotypically resistant to cefoxitin.

**Fig. 3:**
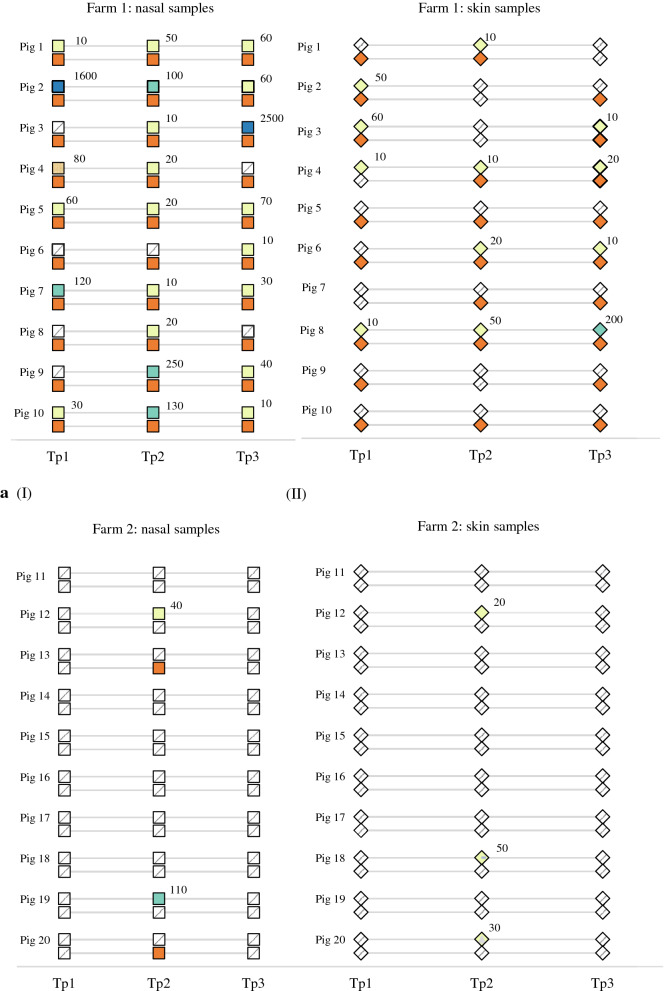

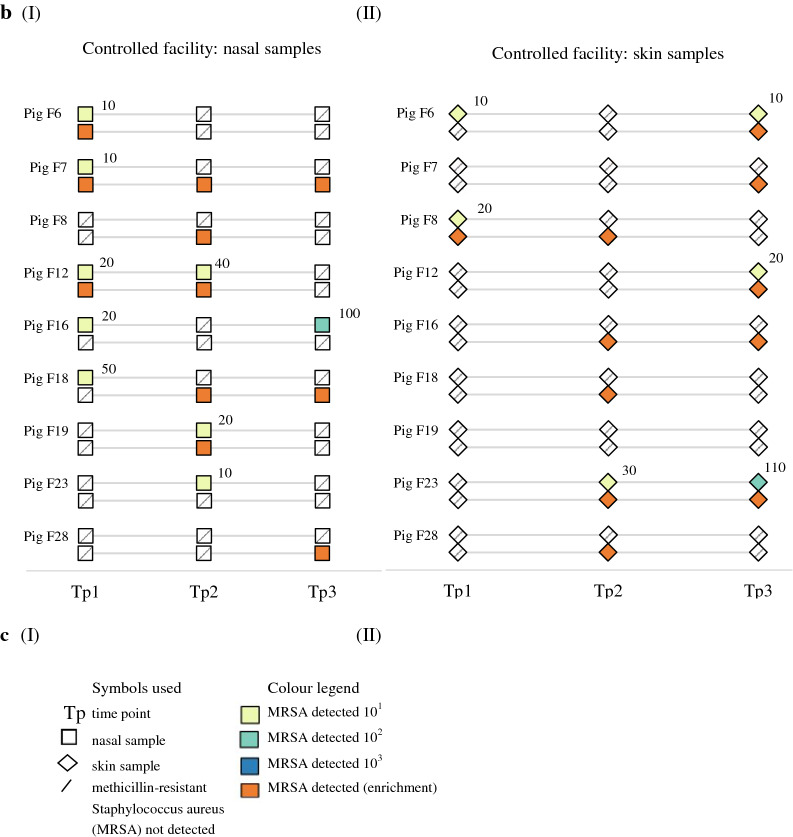
Findings of methicillin-resistant *Staphylococcus aureus* (MRSA) in individual pigs per sampling time point in nasal samples (I) and skin samples (II) on farm 1 (**a**), farm 2 (**b**) and in the controlled facility (**c**). The first row shows quantitative results (figures next to coloured squares in CFU/swab) and the second row shows the results of enrichment

On farm 1 in study A, MRSA was detected in the nares of all 10 pigs at all three time points (Fig. 3aI) but MRSA was detected on the skin at all time points in only five pigs (Fig. 3aII). Nasal quantities of MRSA in these pigs varied between 10 and 10^3^ CFU/swab, with 73.9% (17/23) having a quantity of 10 CFU/swab, 17.4% (4/23) a quantity of 10^2^ CFU/swab and 8.7% (2/23) a quantity of 10^3^ CFU/swab (actual CFU counts are depicted in Fig. 3). MRSA quantity in the positive skin samples was 10 CFU/swab in all but one sample, which showed a quantity of 10^2^ CFU/swab. None of the pigs was a high carrier (≥ 10^4^ CFU/swab at all time points) as none of the pigs had quantities higher than 10 CFU/swab in all positive nose or skin swabs.

On farm 2 in study A (Fig. 3b), MRSA was detected only in samples taken at the second time point. No MRSA was detected in any of the animals during the first time point of sampling (a week earlier) or during the third time point of sampling (a week later). In the second sampling, MRSA was found in the nasal samples of four pigs. MRSA was also found on the skin of two of these four pigs and one additional pig. From skin samples, MRSA was found only by direct plating. The two nasal samples that were MRSA-positive by direct plating had quantities of 10 and 10^2^ CFU/swab, while the three skin samples all had quantities of 10 CFU/swab.

In the controlled facility in study B (Fig. 3c), MRSA was found in the nares of all nine pigs at at least one time point. The bacterial quantities ranged from 10 CFU/swab in eight samples to 10^2^ CFU/swab in one sample. MRSA was detected by enrichment in all nasal samples of one pig. MRSA was detected on the skin of eight pigs. It was found by direct plating in six samples of four pigs with quantities of 10 CFU/swab (five samples) and 10^2^ CFU/swab (one sample). There was a decline in the number of pigs positive in nasal samples processed by direct plating (five pigs at the first time point, three at the second and one at the third) and a decline in the number of nasal samples positive by both enrichment and direct plating (three pigs at the first time point, two at the second and none at the third). A trend in the quantities was not observed.

### Most strains belonged to spa type t034

A total of 30 of 89 isolates from farm 1 and 6 of 7 isolates from farm 2 in 2016, and 21 of 36 isolates from the controlled facility and 9 of 9 isolates from farm 1 in 2017, were subjected to WGS as one strain from each farm had been lost prior to sequencing. However, sequencing failed for two isolates from farm 1, and these could not be analysed further. Altogether, 64 sequences were obtained (Fig. 2).

A *spa* type through sequencing of the PCR product was obtained for 33 of the isolates from farm 1 and 2 of the isolates from farm 2 (Table 2; Additional file 2). Except for one isolate from farm 1, all isolates proved to be of *spa* type t034. The one isolate belonged to t1255. BioNumerics could not provide a reliable *spa* type for two of the strains from farm 1 and three of the strains from farm 2. Analysis of the whole genome sequences of the strains from farm 1 and farm 2 resulted in three *spa* types: t011 (n = 19), t034 (n = 13) and t1255 (n = 1). When compared with the PCR results, there was a discrepancy in the results of 16 strains. The strains isolated at the controlled facility all belonged to *spa* type t034 by both PCR and WGS.

**Table 2:** Results for 88 methicillin-resistant *Staphylococcus aureus* (MRSA) strains *spa* typed by polymerase chain reaction (PCR) and/or whole genome sequencing (WGS)

Number of strains		*spa* type (PCR)	*spa* type (WGS)^a^	Sequence type (ST)^a, b^	Resistance genes^a^	Virulence genes^a, c^	SCC*mec* element^a^
Study A							
Farm 1	Farm 2						
13	1	t034	t011	ST398	*mecA*, *blaZ*, *lnu*(B), *lsa*(E), *tet*(M), *tet*(K), *dfrG*	*aur*, *hlgA, hlgB*, *hlgC*	Vc (5C2&5)
2	0	t034	t011	ST398	*mecA*, *blaZ*, *lnu*(B), *lsa*(E), *tet*(M), *dfrG*	*aur*, *hlgA*, *hlgB*, *hlgC*	Vc (5C2&5)
1	0	t034	t1255	ST398	*mecA*, *blaZ*, *str*, *lnu*(B), *lsa*(E), *tet*(M), *tet*(K), *dfrG*	*aur*, *hlgA, hlgB*, *hlgC*	Vc (5C2&5)
8	1	t034	t034	ST398	*mecA*, *blaZ*, *lnu*(B), *lsa*(E), *tet*(M), *tet*(K), *dfrG*	*aur*, *hlgA, hlgB*, *hlgC*	Vc (5C2&5)
1	0	t034	t034	ST398	*mecA*, *blaZ*, *lnu*(B), *lsa*(E), *tet*(M), *dfrG*	*aur*, *hlgA*, *hlgB*, *hlgC*	Vc (5C2&5)
2	1	Failed	t011	ST398	*mecA*, *blaZ*, *lnu*(B), *lsa*(E), *tet*(M), *tet*(K), *dfrG*	*aur*, *hlgA, hlgB*, *hlgC*	Vc (5C2&5)
0	3	Failed / Not sequenced	t034	ST398	*mecA*, *blaZ*, *lnu*(B), *lsa*(E), *tet*(M), tet(K), *dfrG*	*aur*, *hlgA*, *hlgB*, *hlgC*	Vc (5C2&5)
1	0	t034	Unknown	ST398	*mecA*, *blaZ*, *lnu*(B), *lsa*(E), *tet*(M), *tet*(K), *dfrG*	*aur*, *hlgA, hlgB*, *hlgC*	Vc (5C2&5)
7	1	t034	Not whole genome sequenced / sequencing failed				
1	0	t1255	Not whole genome sequenced				
Study B							
Farm 1	Controlled facility						
0	21	t034	t034	ST398	*mecA*, *blaZ*, *lnu*(B), *lsa*(E), *tet*(M), *tet*(K), *dfrG*	*aur*, *hlgA, hlgB*, *hlgC*	Vc (5C2&5)
9	0	Not sequenced	t034	ST398	*mecA*, *blaZ*, *lnu*(B), *lsa*(E), *tet*(M), *tet*(K), *dfrG*	*aur*, *hlgA*, *hlgB*, *hlgC*	Vc (5C2&5)
0	15	t034	Not whole genome sequenced				

^a^The following tools from the Center for Genomic Epidemiology were used: spaTyper 1.0 [35] for *spa* typing, MLST [37] for multilocus sequence typing, ResFinder [38] for resistance gene detection, VirulenceFinder [39] for virulence gene detection and SCC*mec*Finder 1.2 [36] for staphylococcal cassette chromosome *mec* (SCC*mec*) typing

^b^Multilocus sequence type

^c^VirulenceFinder database for MRSA includes virulence genes *hlb*, *hlgABC*, *tst*, *lukED*, *lukFS-PV*, *etAB*, *edinABC*, *aur*, *splABE*, *scn*, *sak*, ACME and enterotoxins A-E, G-O, R, U, Q

All the strains analysed by WGS belonged to ST398 and shared a very similar set of resistance genes and virulence genes (Table 2; Additional files 2, 3). Neither Panton-Valentine leucocidin genes *lukF* and *lukS* nor genes belonging to the human immune evasion cluster, *scn* and *sak*, were found in any of the strains. All strains carried staphylococcal cassette chromosome *mec* (SCC*mec*) subtype Vc(5C2&5) (Table 2).

### Dominating t034 clone found in core genome multilocus sequence typing

**Fig. 4:**
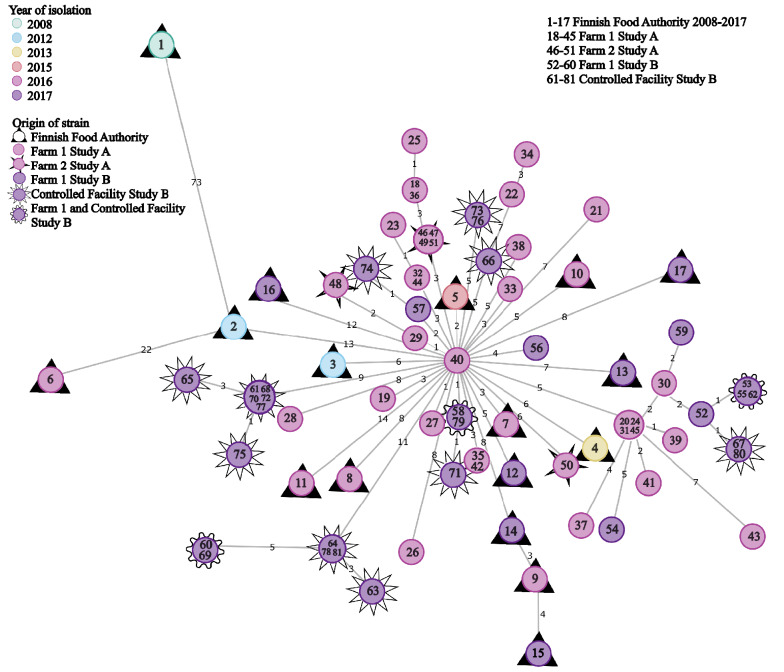
Minimum spanning tree based on sequences of 81 whole genome sequenced methicillin-resistant *Staphylococcus aureus* isolates belonging to *spa* type t034 obtained from Finnish pigs. The tree is based on core genome multilocus sequence typing using 1861 genes. Columns with missing values were removed for calculation. The numbers in the circles represent the strains. The numbers on the connecting lines between the circles represent the allelic difference between the strains. Colours indicate year of isolation. A detailed list of the strains with corresponding numbering can be found in Additional files 2, 4

A minimum spanning tree based on the cgMLST of the 34 sequenced isolates from both farms isolated in 2016, and the 21 from the controlled facility and the 9 from farm 1 in 2017, all showed strains clustering into one cluster, with the minimum allele difference ranging from 0 to 11. When including the 17 strains from the Finnish Food Authority, the strains clustered into the same cluster, except for the first Finnish surveillance strain from 2008, which had a difference of 73 alleles compared with the cluster (Fig. 4). Within the cluster, allele differences ranged from 0 to 22.

Based on the cgMLST analysis, only one clone of t034 was circulating in the pigs sampled on farm 1, and this clone still circulated on the same farm in 2017. The strains isolated on farm 1 in 2016 were very closely related to the strains isolated on farm 2. Nine LA-MRSA strains sequenced by the Finnish Food Authority isolated in 2012, 2013, 2015, 2016 and 2017 were just as closely related to these strains (within 11 alleles).

## Discussion

This study provides new insights into LA-MRSA nasal and skin carriage in pigs. In addition, cgMLST shows closely related strains isolated from pigs both on the studied farms and at the national level during several years, indicating that a highly successful LA-MRSA t034 clone has been circulating in Finnish pigs during 2012–2017. However, repeated introduction of this strain from abroad cannot be ruled out even though imports of live pigs have been at a very low level in Finland. Further studies on global LA-MRSA strains from pigs are warranted.

On farm 1, the samples of all 10 pigs were repeatedly positive. This could either be interpreted as colonization or repeated contamination. MRSA colonization in pigs is known to vary depending on the age of the pigs [16, 17] and the anatomical site [17]. It has been suggested that pigs are not persistently colonized but rather repeatedly contaminated and that this might be an effect of high MRSA loads in the environment and surrounding air. Persistent carriage of MSSA has been associated with higher nasal loads in pigs and removing these high-carriage pigs has been suggested as an MRSA control measure on pig farms [21]. The authors hypothesize that only the high-carrier pigs are true carriers of MRSA and that the other pigs are repeatedly contaminated by these pigs or the environment. In our study, most of the samples from the pigs showed low MRSA loads (< 10^3^ CFU/swab) as did the majority of the samples in the study by Espinosa-Gongora et al. [25] even though differences in methods allow no direct comparison of the quantities between the two studies.

In our study, nine MRSA-positive pigs from farm 1 were moved to a controlled environment. The decline in detectable levels of MRSA in the nasal samples of these pigs might be related to the transfer of the pigs into a controlled environment and therefore reduced MRSA exposure through barn air. As the housing of the pigs in the controlled facility was cleaned and disinfected before the trial, it is unlikely that the air in the controlled facility reached as high levels of MRSA contamination as seen in a barn with a constant high density of LA-MRSA-positive pigs. Ideally, the same pigs would have been sampled on farm in 2016 and in the controlled facility to be certain of MRSA levels in these pigs before the transfer.

When looking at the skin samples from the controlled facility, the picture is less clear. It seems that the number of pigs positive by any of the methods increased from two at the first time point to five at the second and third time points, possibly indicating that MRSA contamination of the environment in the controlled facility increased throughout the trial period as has been seen before [42]. A much longer study period and repeated decontamination of the environment would be necessary to find out whether the bacteria were still able to multiply in the pigs, as LA-MRSA has been shown to survive outside its host in farm dust for weeks [43]. In hindsight, environmental samples would have given important information on the contamination level on the farms compared with the controlled facility. The two-step enrichment method used for study B is known to be less sensitive than the one-step enrichment method used in study A [44] and may have led to false-negative results. It was used, however, to avoid *Enterococcus* spp. overgrowth on the selective plates, which complicated isolation of MRSA in study A.

Farm 2 was known to receive gilts regularly from farm 1. Thus, it was surprising that the sampled pigs on farm 2 did not seem to be repeatedly LA-MRSA-positive. The change in the MRSA status from negative to positive of five pigs seemed to be only temporary. As only pigs from one pen were sampled it is possible that the farm was in fact LA-MRSA-positive but the sampled pigs happened all to be negative at two time points. The LA-MRSA status of an individual pig has been shown to change several times during the production cycle [15, 16]. However, this seems unlikely in this case as most of the pigs were LA-MRSA-negative at all three time points. As the pigs on both farms were sampled by the same person, differences in sampling procedure should not have affected the results. Nevertheless, sampling of live grower pigs without sedation is not easy to standardize and the sampling technique may vary inadvertently.

Subsequent inquiry with the farmer revealed that new pigs from farm 1 had been transported along the aisle next to the pen of the sampled pigs. If the reason for a temporary change in MRSA status was in fact transient contamination of the surroundings with LA-MRSA, it would be interesting to know why LA-MRSA did not survive in these pigs. Identified risk factors for LA-MRSA carriage in pigs include use of antimicrobials [45] and zinc oxide [46], animal trade [47] and a high number of animals [48]. Comparing farms 1 and 2, the use of zinc oxide and a higher number and density of pigs are known risk factors that applied for farm 1 but not farm 2. However, it is impossible to assess their role in this study. Further studies are needed to investigate factors in the environment and the pigs on LA-MRSA-negative farms that may contribute to LA-MRSA-negative status even after repeated introduction.

To our knowledge, the quantity of LA-MRSA in porcine skin samples has not been studied before. In our study, bacterial quantity in skin samples taken behind the ear was at a similar level or lower than in nasal samples. Results for the sensitivity of skin samples compared with nasal samples are conflicting [17, 49, 50]. At herd level and in the slaughterhouse, skin samples have been shown to be more sensitive [49, 50], while at the level of the individual pig, nasal samples have been more sensitive [17]. However, there is no recommended standard for sampling the skin behind the ear and the sampled areas have varied from study to study. As no replicates of the quantitative analysis were performed, the results are only indicative. It should further be noted that the number of pigs sampled on the farms and in the controlled facility is low. Results can therefore not be generalized to the farm level. In addition, with bacterial quantities close to detection level, negative results at the level of the individual pig should be considered with caution.

According to PCR-based *spa* typing results, all typed strains except one were of *spa* type t034, which is the most common ST398 *spa* type in Finnish pig and human samples [13, 14]. Its proportion of LA-MRSA strains in human surveillance samples in Finland has been rising and reached 72% in 2020 [14]. Besides t034, *spa* type t2741 was prevalent in pigs in 2016–2017 but it was not detected on the studied farms [13]. When comparing the results obtained by PCR and WGS, *spa* types were not always assigned correctly by the WGS-based tool, most likely due to the shorter read length of part of the whole genome sequences [35]. Antimicrobial resistance and virulence gene patterns were typical for strains isolated from pigs and there was little to no variation between strains isolated on the two farms and in the controlled facility even between the two years. As only single samples were taken at each time point and single colonies analysed, it is not possible to assess variation within individual pigs.

The detected resistance genes mirror the antimicrobials used in pigs in Finland and on the two farms but are also typical for European LA-MRSA CC398 strains [7]. The most commonly used antimicrobial on Finnish pig farms is penicillin. Other frequently used antimicrobials include amoxicillin, tetracyclines and sulpha-trimethoprim [51, 52]. Although tetracyclines were not in use on the two farms at the time of the study, most of the strains carried two tetracycline resistance determinants, *tet*(M) and *tet*(K), the carriage of which is typical for LA-MRSA CC398 strains [7]. In addition to resistance determinants in these antimicrobial groups, all strains harboured resistance genes *lnu*(B) and *lsa*(E), conveying resistance to lincosamides, pleuromutilins and streptogramin A. The pleuromutilin tiamulin and lincosamide lincomycin are mentioned in the national guideline on use of antimicrobials in pigs as treatment options for a limited range of pig diseases (lincomycin only to be used after susceptibility testing). Sales of these antimicrobials for the treatment of livestock have been at a low level, but species-specific sales information is lacking [53]. A selection of virulence genes was tested and only a few were detected. Neither Panton-Valentine leucocidin encoding genes nor genes of the human immune evasion cluster were detected, which is typical for the livestock-associated clade of MRSA CC398 [54]. All strains harboured SCC*mec* subtype Vc(5C2&5). This was the dominant SCC*mec* subtype in LA-MRSA CC398 in two studies including sequences from several European countries [54, 55]. SCC*mec* Vc(5C2&5) is known to carry heavy metal resistance gene *crzC* and tetracycline resistance gene *tet*(K) in addition to *mecA*. It has been suggested that the use of zinc oxide in pig production may have favoured the selection of strains carrying SCC*mec* Vc [55]. The effects of an EU-wide ban on zinc oxide in pig husbandry as of 26 June 2022 on the prevalence of LA-MRSA CC398 remain to be seen.

The strains were further compared with cgMLST for higher resolution. Sequences of ST398 t034 from the Finnish Food Authority’s surveillance and infection samples were added to see the diversity of strains at the national level. All t034 strains formed one cluster with the exception of the first LA-MRSA strain isolated in Finland. It is known that the evolution rate of *S. aureus* in general [56] and of LA-MRSA in particular is relatively high, with a mutation rate of 1.68 × 10^−6^ or even 2.43 × 10^−6^ base substitutions per site per year for LA-MRSA [57, 58]. Therefore, it was surprising that several LA-MRSA strains sequenced by the Finnish Food Authority over a time period of five years were so closely related to the strains isolated on the two farms in 2016. The Finnish Food Authority samples originated from surveillance, screening and infection samples and, theoretically, only two of the slaughterhouse samples could originate from each of the study farms. The genetically closest strain, isolated in 2015, showed a minimum allele difference of two alleles. The results might indicate that this clone is extremely successful and is not under pressure to evolve; however, this would require a lengthier study period and a higher number of samples to be verified. A German study [59] comparing LA-MRSA strains isolated in 2016 found that within-farm variation between t034 isolates was relatively low compared with *spa* types t011 and t2011. However, in the German study, variation between farms was high and strains mostly clustered according to the individual farms, with allele distances of 92 and 106 between the clusters. Another study comparing LA-MRSA isolates from animals, humans, milk and milking equipment on dairy farms found higher divergence between t034 isolates within farms, with isolates clustering into several clusters within one farm [60].

A recent study on the within-host evolution of MRSA found a median of 5.0 allele variants/individual/year in cgMLST in persistent human carriers of community-acquired MRSA strains (non-CC398) sampled during several years [61]. In our study, the allele differences between the farm and controlled facility strains within the cluster and originating from different pigs were comparable to this variation. The genetic homogeneity of pigs on a pig farm may favour the slow evolution of the strains despite changing host animals. Core-genome-based approaches are used increasingly in outbreak situations when conventional molecular typing, such as *spa* typing, does not provide enough resolution to identify the source of the outbreak and the patients involved. Based on this study it seems that for LA-MRSA t034 strains in Finland, cgMLST may not provide enough resolution to find the source of the strains, and methods based on single nucleotide polymorphisms such as maximum likelihood analysis might prove more useful. The strains isolated on farm 2 were very closely related to those from farm 1 but it is impossible to deduce whether they originated there.

## Conclusions

This is the first study investigating quantities of LA-MRSA in both nasal and skin samples from pigs and examining the genetic diversity of the strains. Previous findings have demonstrated that LA-MRSA-positive pigs are often contaminated rather than colonized. Also, in this study, nasal carriage rates showed a decline in the controlled facility, while there was no difference in skin carriage rates. However, more research is needed involving also environmental samples to further clarify this issue.

The cgMLST analysis of the strains, including also strains from Finnish Food Authority surveillance and infection samples, further suggests that strains of the most common *spa* type t034 in Finnish pigs are very stable. Further studies should look more closely into the reasons which give this clone an advantage compared with the other clones circulating and whether LA-MRSA t034 strains isolated from humans in Finland are as closely related to this clone. Finding the reasons why the clone is so successful may help to control LA-MRSA in pig herds.

## Supplementary Information

**Additional file 1:** Details of methicillin-resistant *Staphylococcus aureus* strains isolated from Finnish Food Authority surveillance and infection samples.

**Additional file 2:** Details of all methicillin-resistant *Staphylococcus aureus* strains isolated in study A.

**Additional file 3:** Details of all methicillin-resistant *Staphylococcus aureus* strains isolated in study B.

**Additional file 4:** Accession numbers of sequences stored in the European Nucleotide Archive under study accession number PRJEB46139 (secondary accession ERP130345).

## Data Availability

Data are contained within the article and the supplementary material. The genomic sequences of the bacteria are available in the European Nucleotide Archive, study accession number PRJEB46139. The individual accession numbers for each strain can be found in Additional file 4.
